# Magneto-Optical Study of Defect Induced Sharp Photoluminescence in LaAlO_3_ and SrTiO_3_

**DOI:** 10.1038/srep33145

**Published:** 2016-09-13

**Authors:** Soumya Sarkar, Surajit Saha, M. R. Motapothula, Abhijeet Patra, Bing-Chen Cao, Saurav Prakash, Chun Xiao Cong, Sinu Mathew, Siddhartha Ghosh, Ting Yu, T. Venkatesan

**Affiliations:** 1 NUSNNI-NanoCore, 5A Engineering Drive 1, National University of Singapore, 117411, Singapore; 2 NUS Graduate School for Integrative Sciences and Engineering, 28 Medical Drive, National University of Singapore, 117456, Singapore; 3 Department of Physics, 2 Science Drive 3, National University of Singapore, 117542, Singapore; 4 Division of Physics and Applied Physics, School of Physics and Mathematical Sciences, Nanyang Technological University, 637371, Singapore; 5 Department of Electrical and Computer Engineering, National University of Singapore, 117576, Singapore; 6 Department of Materials Science and Engineering, National University of Singapore, 117575, Singapore

## Abstract

Strongly correlated electronic systems such as Transition Metal Oxides often possess various mid-gap states originating from intrinsic defects in these materials. In this paper, we investigate an extremely sharp Photoluminescence (PL) transition originating from such defect states in two widely used perovskites, LaAlO_3_ and SrTiO_3_. A detailed study of the PL as a function of temperature and magnetic field has been conducted to understand the behavior and origin of the transition involved. The temperature dependence of the PL peak position for SrTiO_3_ is observed to be opposite to that in LaAlO_3_. Our results reveal the presence of a spin/orbital character in these transitions which is evident from the splitting of these defect energy levels under a high magnetic field. These PL transitions have the potential for enabling non-contact thermal and field sensors.

Transition metal oxides (TMO) exhibit a variety of novel properties^1^ like high dielectric constant, ferromagnetism, ferroelectricity, ferro-elasticity, multiferroicity, colossal magnetoresistance and superconductivity. These properties of TMO systems can be attributed to strong electron correlations which constrain the number of electrons at a given lattice site and induce a local interaction of charge, spin and orbital degrees of freedom. Among these, TMO based Perovskites are a special type of complex oxides which can be described by the generalized formula AMO_3_, where A and M are cations and the transition metal ion (M) is surrounded by six oxygen ions producing a crystal electric field on M with cubic symmetry. Due to the strongly correlated nature of such perovskites, understanding the fundamentals of orbital physics and various quasiparticle transitions in these systems is extremely important. Further, defect engineering at these perovskite interfaces has been demonstrated by various interface phenomena with potential for tailor-made novel properties^2^,^3^,^4^,^5^,^6^,^7^,^8^.

One way to probe such defect states is by observing the photoluminescence emerging from them. We investigated the photoluminescence (PL) of some of the common transition metal oxides like LaAlO_3_, SrLaAlO_4_, LSAT [(LaAlO_3_)_0.3_-(Sr_2_AlTaO_6_)_0.7_)] and SrTiO_3_, which show a very strong photoluminescence in the near-infrared energy band from 1.5 eV to 1.7 eV [Supplementary Fig. (1)]. This energy range is particularly interesting as various solid state lasing sources like the historic Ruby laser and AlGaAs semiconductor laser exhibit strong emissions close to this band^9^,^10^,^11^. In this work, we have narrowed down our study on LaAlO_3_ and SrTiO_3_, as these two materials have an extremely sharp PL with a full width at half maximum (FWHM) of as low as 1 nm. In the case of Ruby, which is essentially Cr doped in Al_2_O_3_, presence of strong and sharp PL lines (tunable with temperature and pressure) has been exploited as temperature and pressure sensors^12^,^13^. A comparable behavior of PL lines in our study with temperature and magnetic field in the LaAlO_3_/SrTiO_3_ system may open up similar possibility. It should also be noted that the above mentioned materials are commonly used as substrates for thin film growth in oxide electronics.

Although, there have been some initial studies^14^,^15^,^16^ on the photoluminescence of these interesting materials, a detailed understanding of the fundamental physics of this near-infrared luminescence in particular and its possible implications is unavailable. For LaAlO_3_, the strong PL at 1.69 eV has been suspected to be due to a crystal anti-site^17^ while in SrTiO_3_ the PL at 1.56 eV is considered to be a transition due to a Frenkel-like exciton^14^. In the present work, we have performed a detailed study of this luminescence as a function of temperature and magnetic field. The magneto-luminescence study would help us probe the presence of potential spin-orbit interactions in these materials. Particle Induced X-Ray Emission (PIXE) Spectroscopy has been performed on these samples to investigate the presence of possible magnetic impurities. Our findings support, complement and further extend the information available in published literature. We believe that the inevitable presence of defects and/or defect induced quasiparticles gives rise to certain energy levels possessing spin-orbit coupled total angular momentum states, which could contribute to this unusual mid-gap luminescence in these materials. With an appropriate engineering of these states, it may be possible to tailor the various novel properties of these oxides for next generation magneto-optic sensors and detectors.

## Results and Discussion

**Figure 1:**
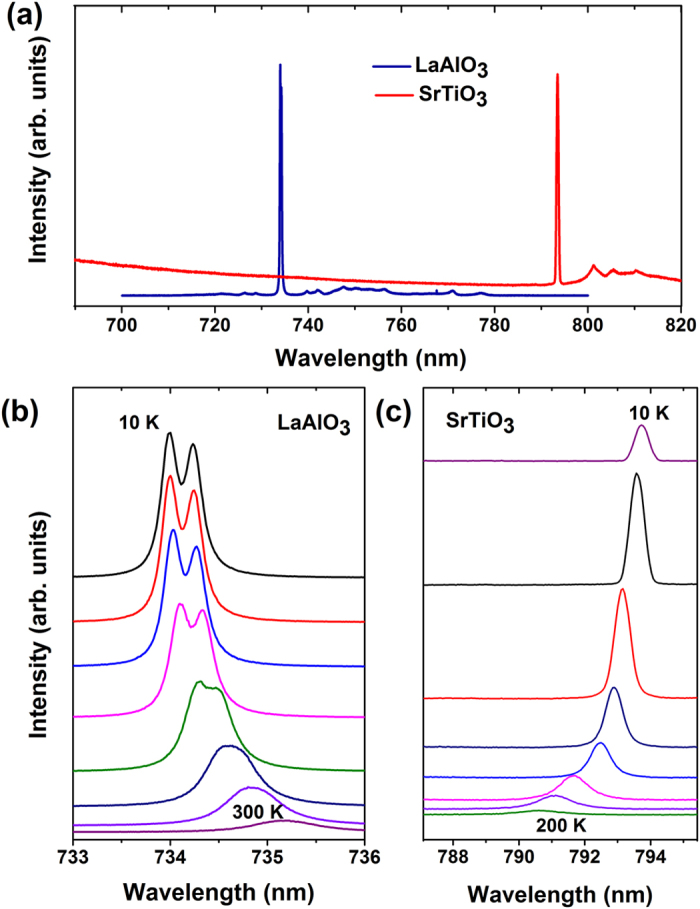
(**a**) Ultra-Sharp PL lines of LaAlO_3_ and SrTiO_3_ have been shown to exist in the near infrared energy band from 1.5 eV to 1.7 eV at 40 K. The evolution of the PL spectra for LaAlO_3_ and SrTiO_3_ for various temperatures have been shown in (**b**,**c**) respectively. The two sub-peaks in LaAlO_3_ is due to its twinning^17^. It is interesting to note the opposite trends in the peak shifts as a function of temperature.

Figure 1(a) shows the sharp PL lines of LaAlO_3_ and SrTiO_3_ in the near IR energy range of 1.5–1.7 eV and their temperature dependence behavior is summarized in the following figures. Figure 1(b) shows the primary peak of LaAlO_3_ at 734 nm (the two components are related to the twinning in LaAlO_3_^17^) which shows a regular red shift with temperature and a decrease in its intensity as we increase the temperature from 10 K to 300 K. Interestingly, the trend observed for the PL peak position as a function of temperature for SrTiO_3_ is anomalous [Fig. 1(c)], the details of which have been discussed later. The anomalous intensity profile of the PL peak is attributed to the two well-known phase transitions in SrTiO_3_, the paraelectric to ferroelectric transition near 35 K and the structural tetragonal to cubic phase transition at 105 K^16^. Both of these luminescent peaks originate due to transitions from mid-gap states, evident from the energy of these transitions at 1.69 eV for LaAlO_3_ and 1.56 eV for SrTiO_3_. Such narrow mid-gap states are often formed as a result of anti-site defects or the presence of impurities^17^,^18^,^19^,^20^,^21^,^22^.

**Figure 2:**
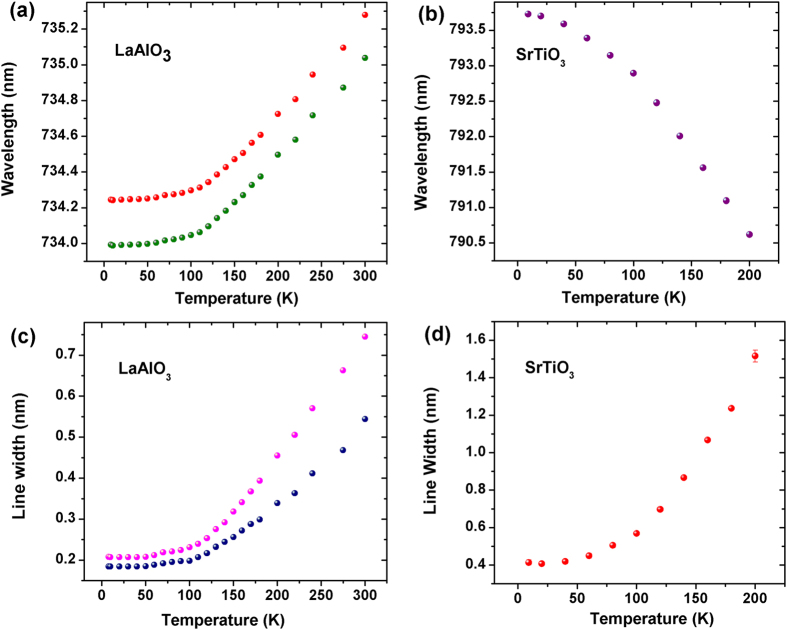
(**a**,**b**) Shows the variation of PL peak position with temperature for LaAlO_3_ and SrTiO_3_. The LaAlO_3_ peaks show a conventional red shift up to 1 nm with increasing temperature while the SrTiO_3_ peak behaves anomalously and has a blue shift of approximately 3 nm. Sub-figures (**c**,**d**) shows the variation of PL linewidth with temperature for LaAlO_3_ and SrTiO_3_. The thermal broadening in case of LaAlO_3_ is almost 0.3 nm–0.5 nm (for both the twins) and approximately 1.2 nm for SrTiO_3_.

Figure 2(a,b) show the detailed variation of the peak position of the luminescence with temperature for LaAlO_3_ and SrTiO_3_, respectively. For LaAlO_3_, the peak shifts to higher wavelengths or lower energy states as we increase the temperature from 10 K to 300 K. The shift in the peak position as seen from the graph is around 1 nm [2.6 meV]. This regular red shift is expected for most systems according to the conventional temperature dependence of photoluminescence. Increasing temperature causes lattice expansion which reduces the interaction energy between the various quasiparticles involved in the transitions thereby shifting the luminescence position to lower energy levels^23^. However, the temperature dependence of the peak position for SrTiO_3_ is anomalous as it shows an interesting ‘blue shift’ with increasing temperature *i.e.* the PL position shifts to higher energy levels by 3.1 nm [6.4 meV] from 10 K to 200 K. Beyond this, the peak intensity attenuates due to phonon contributions at higher temperature. This anomalous shift may be attributed to the ‘pushing effect’^24^,^25^ which occurs when the terminal energy levels are coupled with the electronic states of a neighboring higher energy manifold, not involved in the transition. These separate energy levels have an interaction which causes the luminescent transition levels to be pushed away from each other with increasing temperature, causing the blue shift. The variation of linewidth with temperature is shown in Fig. 2(c) (for LaAlO_3_) and 2(d) (for SrTiO_3_). As evident from the graph the linewidth of the PL peak for both LaAlO_3_ and SrTiO_3_ appears to be increasing with temperature which is attributed to thermal broadening. The linewidth increases by 1.2 nm (SrTiO_3_) and 0.3–0.5 nm (LaAlO_3_) from low temperature to high temperature.

**Figure 3:**
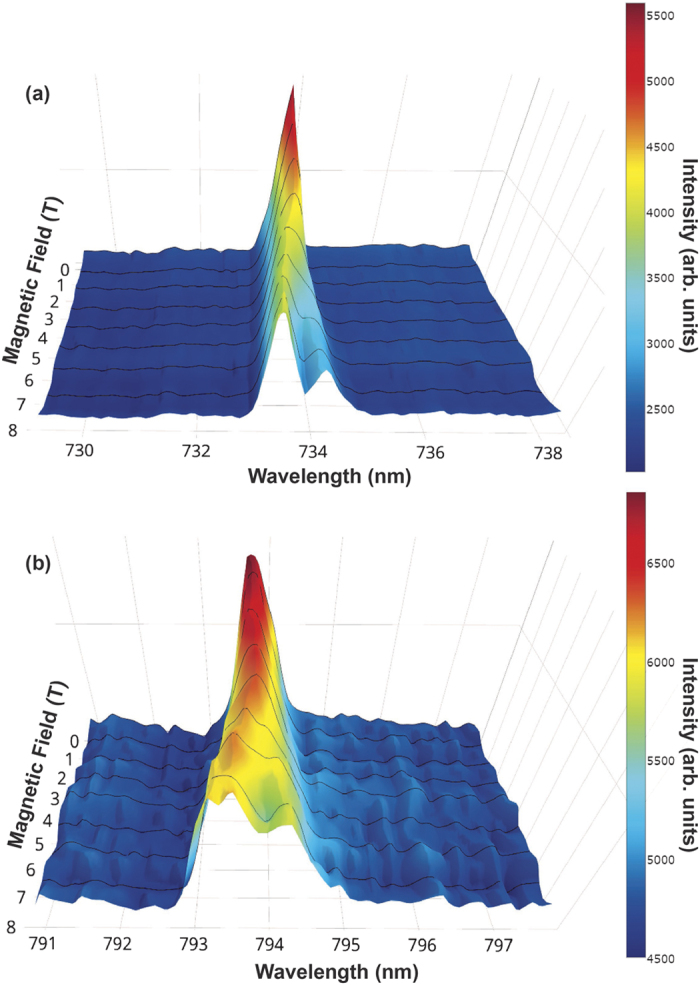
Splitting of the PL peaks in presence of magnetic field (**a**) LaAlO_3_ (**b**) SrTiO_3_. It can be clearly seen that as we increase the magnetic field from zero to 8T, the peaks split into two sub-peaks.

As discussed earlier the origin of this luminescence in these materials could be due to the presence of defects (*e.g.*, Schottky-like or Anti-site) or the presence of impurities (*e.g.*, Frenkel-like)^18^,^19^,^20^. Both types of defects may give rise to some spin and/or orbital states. One way to probe the nature of these spin/orbital states is to investigate their response in the presence of magnetic field which is shown in Fig. 3. For both LaAlO_3_ [Fig. 3(a)] and SrTiO_3_ [Fig. 3(b)], we see a broadening and ultimately splitting into two distinct components as we increase the magnetic field. This splitting clearly suggests that the luminescence involves magnetic degrees of freedom, *e.g.* spin and/or orbital states.

It has been reported earlier by Chen *et al*.^17^ that for LaAlO_3_ the formation of local La_Al_ anti-site defects or cationic interstitials^21^,^22^ result in quasi-degenerate defect levels at ~1.6 eV^17^. In these levels, the spin-zero La^3+^ interacts with such Schottky like defects to produce a defect complex with finite angular momentum. The formation of such a defect complex may be triggered due to the hybridization of the empty La *‘d’* or *‘f’* orbitals with these defects. On application of magnetic field this finite angular momentum state shows splitting. Such field sensitive transitions originating from defect states in a polar oxide layer like LaAlO_3_ have been reported by Lu W. M. *et al*.^26^. In this context, it should be noted that these twin peaks as shown in Fig. 1(b) can be resolved only by using high resolution gratings. The magnetic field dependent study conducted in the present work was done with a lower resolution grating (Δλ > 0.2 nm) and hence the quasi-degenerate levels at 1.6 eV are not resolved at zero magnetic field. However, with application of magnetic field this composite level splits into two indicating the presence of a spin character S = 1/2. As we increase the magnetic field we see a linear increase in the separation of these two energy levels confirming the Zeeman effect. PIXE measurement on LaAlO_3_ substrates (available from different commercial suppliers) did not show any magnetic impurities at its lowest detection limit [Supplementary Fig. (2a)] ruling out the possibility that magnetic impurities could have any role in this PL.

For SrTiO_3_, it has been reported earlier that this strong PL could be due to a Frenkel exciton, whose electronic structure represents a tightly bound electron hole pair, screened by the optical dielectric constant of SrTiO_3_^14^,^16^. But this alone cannot explain the splitting of the PL in presence of magnetic field. We have quantified by PIXE that SrTiO_3_ shows the presence of Fe and Cr impurities [Supplementary Fig. (2b)]. The presence of Cr impurities is also suggested by previous studies^14^ where an enhancement of this luminescence peak was observed in Cr doped SrTiO_3_. We thus believe that these magnetic impurities create a bound Frenkel like defect state, which on application of magnetic field loses its degeneracy and shows splitting into two components.

**Figure 4:**
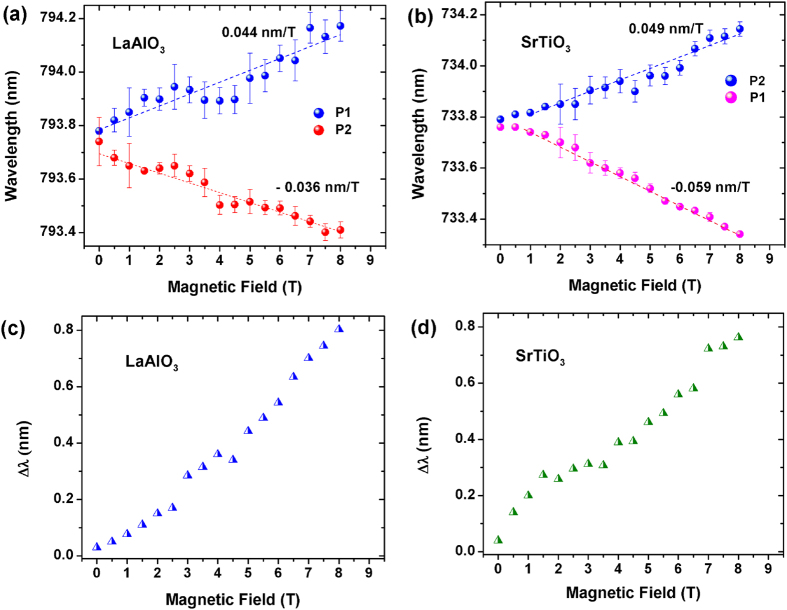
Variation of Peak Position with Magnetic Field for (**a**) LaAlO_3_ and (**b**) SrTiO_3_ has been plotted from 0 T to 8 T. The difference in the peak positions as a function of magnetic field is plotted for (**c**) LaAlO_3_ and (**d**) SrTiO_3._ We see a clear linear behaviour of this peak shift with magnetic field.

The shift of the peak positions on account of splitting in the presence of magnetic field has been illustrated in Fig. 4(a,b). For LaAlO_3_ [Fig. 4(a)] the peak starts splitting with an applied field and as we increase the field to 8T two distinct peaks (P1 and P2) form which are 0.8 nm (~1.9 meV) apart. Similarly, for SrTiO_3_, [Fig. 4(b)] at 8T the energy difference between the two peaks (P3 and P4) is nearly 0.7 nm (~1.4 meV). The splitting of these sharp PL peaks in presence of a magnetic field clearly indicates that these defect have local magnetic moments which are estimated to be ~2.04 μ_B_ for LaAlO_3_ and ~1.51 μ_B_ for SrTiO_3_ (the absolute values of these moments should not be compared because both this transitions have completely different origins). The difference between the peak positions (Δλ) for P1 and P2 in LaAlO_3_ and P3 and P4 in SrTiO_3_ are plotted in Fig. 4(c,d). It is extremely interesting to observe that for both these materials Δλ shows a linear trend with increasing magnetic field which can be exploited to create non-contact PL based magnetic field sensors.

In conclusion, we have performed temperature and magnetic field dependent PL measurements on LaAlO_3_ and SrTiO_3_. Our experiments provide the presence of defect induced PL transitions possessing spin/orbital characters which under magnetic field undergo Zeeman splitting. Such magnetic states being present in widely used substrate materials (LaAlO_3_ and SrTiO_3_) could possibly give rise to a new understanding of novel phenomena occurring at the interface of these oxides and thin films grown on them. Also, the sharp PL lines emerging from these materials show an interesting temperature and magnetic field dependence which could be exploited for developing non-contact temperature and magnetic field sensors. We believe that our observations encourage further studies to understand the possible effects of such defect levels and if appropriately engineered these states may be useful for advanced photonic device and laser applications.

## Methods/Experimental Procedure

The samples examined are commercially available single crystal substrates of LaAlO_3_ and SrTiO_3_ (Crystec, GmbH, Germany and MTI Corp. USA), about 5 mm × 5 mm area and 0.5 mm thick with both sides polished. Photoluminescence measurements were done using a JY Horiba LabRAM HR Evolution Raman Spectrometer coupled with an air cooled CCD. All the data have been recorded at an excitation wavelength of 514.5 nm from a Lexel SHG 95 Argon Ion Laser. The spectra were collected at periodic temperature intervals from 10 K to 300 K to understand the temperature dependence using an Advanced Research Systems Inc. compressed helium based closed cycle refrigerator coupled with the above spectrometer. Magnetic field-dependent measurements were performed using an attoCube superconducting magnet coupled with a WiTec Raman spectrometer with a 532 nm laser line as the source of excitation. To identify and quantify the contaminants in these single crystal substrates, we have conducted Particle Induced X-ray emission technique (PIXE) by using 2 MeV alphas/protons and detected X-ray’s by using Si (Li) detector.

## Additional Information

**How to cite this article**: Sarkar, S. *et al*. Magneto-Optical Study of Defect Induced Sharp Photoluminescence in LaAlO_3_ and SrTiO_3_. *Sci. Rep.* **6**, 33145; doi: 10.1038/srep33145 (2016).

## Supplementary Material

Supplementary Information
