# Supplementary material for: Magneto-Optical Study of Defect Induced Sharp Photoluminescence in LaAlO3 and SrTiO3
Source: Sci Rep. 2016 Sep 13;6:33145. doi: 10.1038/srep33145 (PMC5020611; doi:10.1038/srep33145)
Supplement: Supplementary Information [file srep33145-s1.pdf]

SUPPLEMENTARY INFORMATION

Magneto-optical Study of Defect Induced Sharp Photoluminescence in $LaAlO_3$ and $SrTiO_3$

Soumya Sarkar[1, 2], Surajit Saha[1,3,§], M. R. Mothapotula[1,3], Abhijeet Patra[1], Bing-Chen Cao[4], Saurav Prakash[1,2], Chun Xiao Cong[4, ⊥], Sinu Mathew[1],Siddhartha Ghosh[1], Ting Yu[4], T. Venkatesan[1, 2,3,5,6]

1. NUSNNI-NanoCore, 5A Engineering Drive 1, National University of Singapore, Singapore 117411
2. NUS Graduate School for Integrative Sciences and Engineering, 28 Medical Drive, National University of Singapore, Singapore 117456
3. Department of Physics, 2 Science Drive 3, National University of Singapore, Singapore 117542
4. Division of Physics and Applied Physics, School of Physics and Mathematical Sciences, Nanyang Technological University, Singapore 637371
5. Department of Electrical and Computer Engineering, National University of Singapore, Singapore 117576
6. Department of Materials Science and Engineering, National University of Singapore, Singapore 117575

§ Presently at Indian Institute of Science Education and Research, Bhopal, Madhya Pradesh, 462066, India

⊥ Presently at School of Information Science and Technology, Fudan University, Shanghai 200433, People's Republic of China

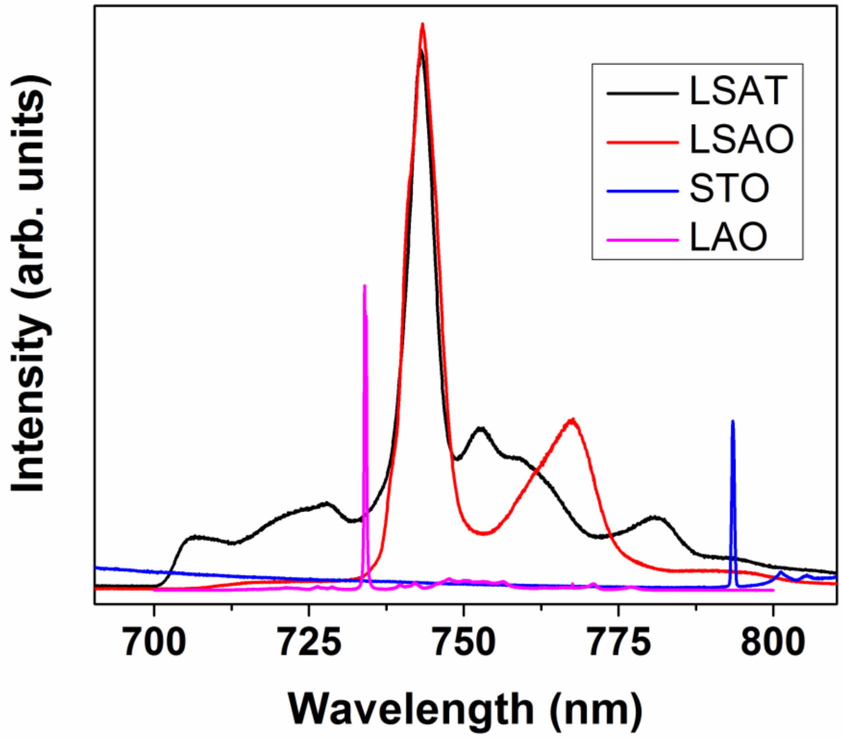

Supplementary Fig S1. PL of LAO, STO, LSAO, LSAT

Common transition metal oxides like LAO ($LaAlO_3$), LSAO ($SrLaAlO_4$), LSAT [$(LaAlO_3)_{0.3}$-$(Sr_2AlTaO_6)_{0.7}$)] and STO ($SrTiO_3$) have a strong photoluminescence in the near infrared energy band of 1.5 to 1.7 eV. Out of these, the PL emerging from $LaAlO_3$ and $SrTiO_3$ is extremely sharp and thus interesting. Fig S1 shows the above mentioned PL at 10 K.

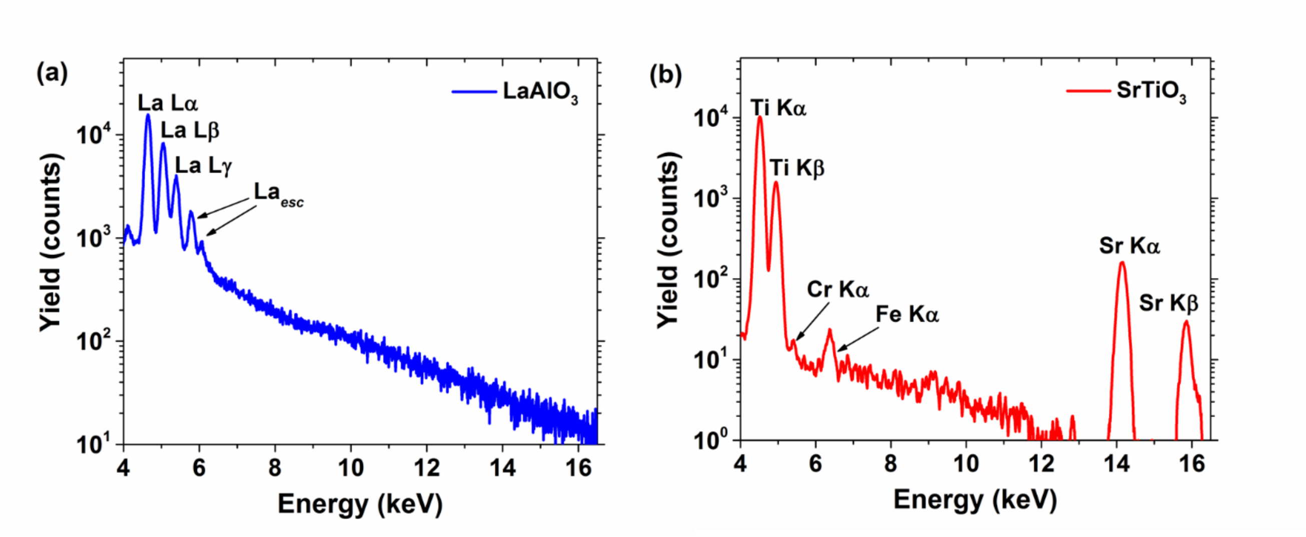

Supplementary Fig S2. PIXE spectra for (a) $LaAlO_3$ and (b) $SrTiO_3$ single crystal substrates.

To investigate the presence of impurities, we have conducted Particle Induced X-ray Emission technique on $LaAlO_3$ and $SrTiO_3$ single crystal substrates from multiple suppliers (Crystec, GmbH, Germany and MTI Corp. USA). No impurities were detected for $LaAlO_3$ upto the lowest detection limit (~1 ppm) of PIXE, $SrTiO_3$ however showed the presence of magnetic impurities Fe and Cr. We have used Fe and Cr standard samples to quantify the contaminants in the substrates and their values are 160 ppm and 7 ppm respectively. All these contaminants are in interstitial sites, confirmed by collecting PIXE spectra under channeling alignment.
